# Comparison of Synthesized Microstructured and Commercial FePO_4_ as Precursors for High-Performance LiFePO_4_/C Cathode Materials

**DOI:** 10.1021/acsomega.6c02734

**Published:** 2026-07-11

**Authors:** Leandro Alves dos Santos, Elton Torres Zanoni, Gabriella M. V. Dias, Sofia Sestito Dias, Francisca E. R. Oliveira, George C. Santos, Frederico F. Taves, Tatiana Araujo De Souza Martins, Rodrigo V. Queiroz, Heverson R. Freitas, Guilherme Panini, Adler de Souza, Agne R. Carvalho-Jorge, Marcos A. C. Berton

**Affiliations:** † 715867 SENAI Institute for Innovation in Electrochemistry, Curitiba, Paraná 82590-300, Brazil; ‡ Federal University of Paraná, Department of Chemistry, Polytechnic Center, P.O. Box 19032, Curitiba 81531-980, Paraná, Brazil; § 251129 Centrais Elétricas Brasileiras SA, Rio de Janeiro 20071-003, Rio de Janeiro, Brazil

## Abstract

In recent years, lithium iron phosphate (LiFePO_4_, LFP) has attracted considerable attention as a cathode material for lithium-ion batteries due to its thermal stability, long cycle life, and low cost. The principal objective of this work was to evaluate the influence of different iron phosphate precursors on the synthesis and electrochemical performance of LiFePO_4_/C prepared by a solid-state route. A microstructured FePO_4_ obtained by controlled precipitation (FP-S) was compared with two commercially available FePO_4_ samples (FP-B1 and FP-B2) and with a synthesis route based on FeSO_4_ (FS). All materials showed the formation of phase-pure olivine LiFePO_4_, as confirmed by X-ray diffraction (XRD). However, significant differences in particle morphology and crystallinity were observed depending on the precursor source. The material derived from FP-S presented a more homogeneous particle size distribution (2–6 μm) and lower degree of agglomeration compared to the samples obtained from commercial phosphates. Electrochemical performance was evaluated under identical conditions. The best result, in terms of discharge capacity, was 158 mAh/g at 0.1C for the material derived from FP-S. The improved electrochemical response observed for the material prepared from the synthesized FePO_4_ indicates that precursor particle size control plays an important role in defining the final microstructure and electrode kinetics. These results suggest that laboratory-designed FePO_4_ precursors can provide better performance consistency than commercially available phosphates, where particle size distribution and morphological control are not well-defined.

## Graphical abstract

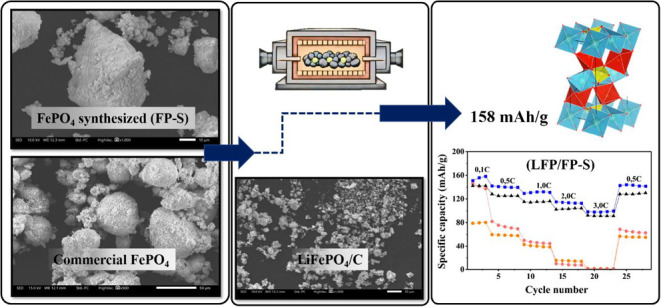

## 1 Introduction

Lithium iron phosphate (LiFePO_4_, LFP) is one of the main cathode materials used in lithium-ion batteries for stationary and large-scale energy storage applications.^1–3^ Its widespread use is associated with low cost, thermal stability, long lifespan, and a theoretical capacity of 170 mAh/g.^4–6^ Furthermore, LFP exhibits a stable voltage plateau at approximately 3.4–3.5 V versus Li^+^/Li and a robust olivine structure that undergoes minimal distortion during lithium insertion and extraction.^7^

Despite these advantages, LFP has its own limitations. First, its intrinsic electronic conductivity is low, which restricts charge transport within the electrode. Second, the diffusivity of lithium ions in the olivine structure is moderate, limiting performance at high charge/discharge rates. Third, particle agglomeration during synthesis can reduce the effective surface area and increase diffusion paths. For these reasons, strategies such as particle size reduction, morphology control, and coating with conductive carbon are commonly used to improve the electrochemical performance.

LiFePO_4_ can be synthesized by various methods, including solid-state reaction, hydrothermal processing, sol–gel routes, and spray pyrolysis.^8–10^ Among these, the solid-state method is widely used because it is simple and scalable. In this route, different iron sources, such as FePO_4_, FeSO_4_, Fe_2_O_3_, and iron oxalate, can be used. When FePO_4_ is the precursor, a carbothermic reduction step under an inert atmosphere is necessary to convert Fe^3+^ to Fe^2+^, while simultaneously forming a conductive carbon coating from an organic carbon source.^11,12^

The nature of the iron precursor influences particle growth, reduction kinetics, and final morphology.^13,14^ Commercial FePO_4_ is commonly used in laboratory and industrial processes. However, their particle size distribution and microstructural characteristics are often not well-defined. This lack of control can affect phase formation, agglomeration behavior, carbon coating organization, and consequently, the electrochemical performance of the resulting LiFePO_4_/C material.^15,16^ Although several studies report different synthesis routes for LFP, few works systematically compare laboratory-synthesized iron phosphates to commercially available batches under identical processing conditions.

The aim of this work was, therefore, to evaluate the influence of different iron phosphate precursors on the synthesis and electrochemical performance of LiFePO_4_/C prepared via a solid-state route. This study is motivated by the lack of control over the physicochemical properties of commercially available FePO_4_ and the limited number of studies comparing different precursors under the same synthesis conditions. In this work, LFP was synthesized from a microstructured FePO_4_, obtained by controlled precipitation, and was compared to two commercial FePO_4_ samples and with a route based on FeSO_4_. The structural, morphological, and electrochemical properties of the resulting materials were analyzed in order to determine the extent to which controlling the particle size of the precursor affects the formation of LiFePO_4_ and battery performance.

## 2 Experimental Section

### 2.1 Materials

Iron phosphate (FePO_4_·2H_2_O) batch 1 and batch 2 (SDBB1988), *N*-methyl-2-pyrrolidone (NMP), were purchased from Sigma-Aldrich Chemie GmbH (Schnelldorf, Germany); iron sulfate (FeSO_4_·7H_2_O), ammonium dihydrogen phosphate (NH_4_H_2_PO_4_), and ammonium hydroxide (NH_4_OH) were from Dinâmica Química Contemporânea Ltd.a. (Indaiatuba, São Paulo, Brazil), iron nitrate (Fe­(NO_3_)_3_·9H_2_O) and d-glucose were purchased from Synth (Diadema, São Paulo, Brazil); lithium carbonate (Li_2_CO_3_, battery grade) was obtained from Companhia Brasileira de Lítio (Araçuaí, Minas Gerais, Brazil); commercial lithium iron phosphate (LiFePO_4_), conductive carbon Super P, and poly­(vinylidene fluoride) (PVDF) were from Gelon (Guangdong, China); lithium disc for coin cell (99.9%, ϕ15.6 × 1.0 mm) was purchased from Tob Machine (Xiamen, China); aluminum foil was a donation from Companhia Brasileira de Alumínio (São Paulo, Brazil).

Throughout the paper, both the precursors and the LiFePO_4_ obtained will be referred to by their respective acronyms, which can be found in Table 1.

**Table 1:** List of Acronyms Used in This Work

entry	name	acronym
1	synthesized FePO_4_	FP-S
2	commercial FePO_4_ (batch 1)	FP-B1
3	commercial FePO_4_ (batch 2)	FP-B2
4	LFP obtained using synthesized FePO_4_	LFP/FP-S
5	LFP obtained using commercial FePO_4_ from batch 1	LFP/FP-B1
6	LFP obtained using commercial FePO_4_ from batch 2	LFP/FP-B2
7	LFP obtained using FeSO_4_	LFP/FS
8	commercial LFP used as reference	LFP/R

### 2.2 Preparation of FePO_4_·2H_2_O

FePO_4_·2H2O was prepared according to procedures described elsewhere previously..^17,18^ First, iron­(III) nitrate nonahydrate (50.5 g, Fe­(NO_3_)_3_·9H_2_O) and ammonium dihydrogen phosphate (14.38 g, NH_4_H_2_PO_4_) were each dissolved in 250 mL of deionized water (18.2 MΩ·cm) in separate 1-L beakers under magnetic stirring. The resulting 0.5 mol L^–1^ solutions were transferred to a 1-L round-bottom flask and stirred at room temperature (23 °C) until complete homogenization. The mixture was then heated to 60 °C, and the pH was maintained between 3.0 and 5.0 by addition of NH_4_OH. The reaction was stirred mechanically for 1 h at 60 °C.

After cooling to room temperature (23 °C), the suspension was filtered under vacuum through a Büchner funnel using Whatman no. 1 filter paper. The solid was washed repeatedly with ultrapure water to remove soluble species and dried at 100 °C for 24 h. The dried material was stored in a desiccator until further use. To obtain anhydrous FePO_4_, the dried precipitate was calcined in a muffle furnace at 720 °C for 2 h.

### 2.3 Synthesis of LiFePO_4_/C

LiFePO_4_/C was prepared by a solid-state carbothermic reduction route using iron precursors with different oxidation states. Three ferric phosphate sources were used, namely FP-S (Table 1, Entry 1), FP-B1 (Table 1, Entry 2), and FP-B2 (Table 1, Entry 3). For the routes using FP-S, FP-B1, and FP-B2, FePO_4_ (12.5 g, 0.0634 mol), Li_2_CO_3_ (3.02 g, 0.0317 mol), and d-glucose (1.55 g, corresponding to 10 wt % relative to the total mass of FePO_4_ and Li_2_CO_3_) were placed in nylon jars containing agate grinding spheres. The mass ratio between spheres and powder was fixed at 5:1. Isopropyl alcohol was added in a 1:1 proportion relative to the mass of the grinding spheres. The mixture was homogenized at 300 rpm for 12 h. The suspension was then centrifuged at 3000 rpm and dried at 70 °C for 24 h. The dried powder was subjected to a second grinding step for 5 min at 100 rpm using a 1:1 mass ratio between balls and powder. For the FeSO_4_·7H_2_O route, the same procedure was followed, with adjustment of reagent quantities to maintain stoichiometric equivalence. FeSO_4_·7H_2_O (17.66 g, 0.0634 mol), Li_2_CO_3_ (2.344 g, 0.0317 mol), and NH_4_H_2_PO_4_ (7.30 g, 0.0634 mol) were used, together with 10 wt % d-glucose relative to the total mass of solids. The homogenized mixtures were calcined in a tubular furnace at 700 °C under an Ar atmosphere. d-glucose acted as both the reducing agent and the carbon source. For LFP/FP-S and LFP/FS, reaction times of 6, 10, and 20 h were investigated. For LFP/FP-B1 and LFP/FP-B2, a reaction time of 10 h was employed.

### 2.4 Characterization

The phase purity and crystalline structure of the samples were determined by X-ray diffraction using Cu Kα radiation (λ = 1.5405 Å) on a D2 Phaser diffractometer (Bruker-AXS) equipped with a Ni filter. Diffraction patterns were collected in the 2θ range from 10 to 90°, with a step size of 0.02° and a counting time of 2 s per step.^19^ The morphology and microstructure of the samples were observed by scanning electron microscopy (SEM) using a JCM-7000 microscope (JEOL) with a tungsten filament electron source. Elemental distribution was analyzed by energy-dispersive X-ray spectroscopy (EDS) using the integrated detector. Raman spectra were recorded on a Senterra Raman spectrometer equipped with a 50× objective lens and a 633 nm excitation laser. For the LFP samples, a laser power of 5 mW was used in the spectral range of 50–3700 cm^–1^, with an integration time of 45 s. Spectra were collected at different points to evaluate sample homogeneity. The carbon content of the LFP samples was determined using a LECO CS844 analyzer under an O_2_ flow of 3 L min^–1^. All measurements were performed in triplicate. The elemental composition (Fe, P, and Li) was analyzed by inductively coupled plasma optical emission spectroscopy (ICP-OES) using a Thermo iCAP Duo 6500 instrument after acid digestion. The thermal behavior of FePO_4_ and the LFP precursors was evaluated by thermogravimetric analysis (TGA) under N_2_ atmosphere using a Netzsch STA 449 F5 Jupiter instrument at a heating rate of 10 °C min^–1^ from 30 to 1000 °C.

### 2.5 Electrochemical Measurements

The electrochemical performance of the as-prepared LiFePO_4_/C materials was evaluated using CR2032-type coin cells assembled in half-cell configuration.^20^ For electrode preparation, 18 g of LFP, 1.0 g of Super P conductive carbon, and 1.0 g of PVDF were dispersed in 30 mL of NMP. The mixture was homogenized in a planetary mixer (Gelon, 150 mL jar) at 600 rpm for 4 h until a uniform slurry was obtained. The slurry was cast onto aluminum foil using a film applicator with a 150 μm doctor blade gap and dried under vacuum at 80 °C. Circular electrodes (15 mm diameter) were punched from the dried films. Metal lithium was used as both counter and reference electrode. The cells were assembled in an argon-filled glovebox. Galvanostatic charge–discharge measurements were carried out at 25 °C using a (Neware Co.) battery tester (CT-4000–5 V100 mA) in a voltage window of 2.5–4.2 V vs Li/Li^+^. Before electrochemical testing, the cells were allowed to rest for 12 h. Formation cycles were performed under constant-current/constant-voltage conditions at C/10, with a cutoff current of C/40 during the constant-voltage step. Three complete formation cycles were applied. Rate capability was evaluated by charging at 0.5C to 3.6 V, followed by a constant-voltage step at 3.6 V until the current decreased to C/40. Discharge was performed at 0.5C, 1.0C, 2.0C, 3.0C, and again 0.5C, with a cutoff voltage of 2.5 V. Each rate was applied for five consecutive cycles. Cycling stability tests were conducted for 100 cycles, with charging at 0.5C and discharging at 1.0C within the same voltage window. Electrochemical impedance spectroscopy (EIS) measurements were conducted on an Autolab (302N) potentiostat/galvanostat (Metrohm Co.) using a frequency range of 100 kHz to 100 mHz and an amplitude of 10 mV in half-cells at 50% state of charge (i.e., discharge cutoff voltage of 3.4 V in the third cycle of formation).

## 3 Results and Discussion

Figure 1 shows the XRD patterns of the FePO_4_ before and after calcination. The pattern in Figure 1a corresponds to the precipitated FePO_4_·2H_2_O obtained by controlled precipitation. The diffraction profile exhibits a broad halo and absence of sharp reflections, indicating its predominantly amorphous character.

**Figure 1:**
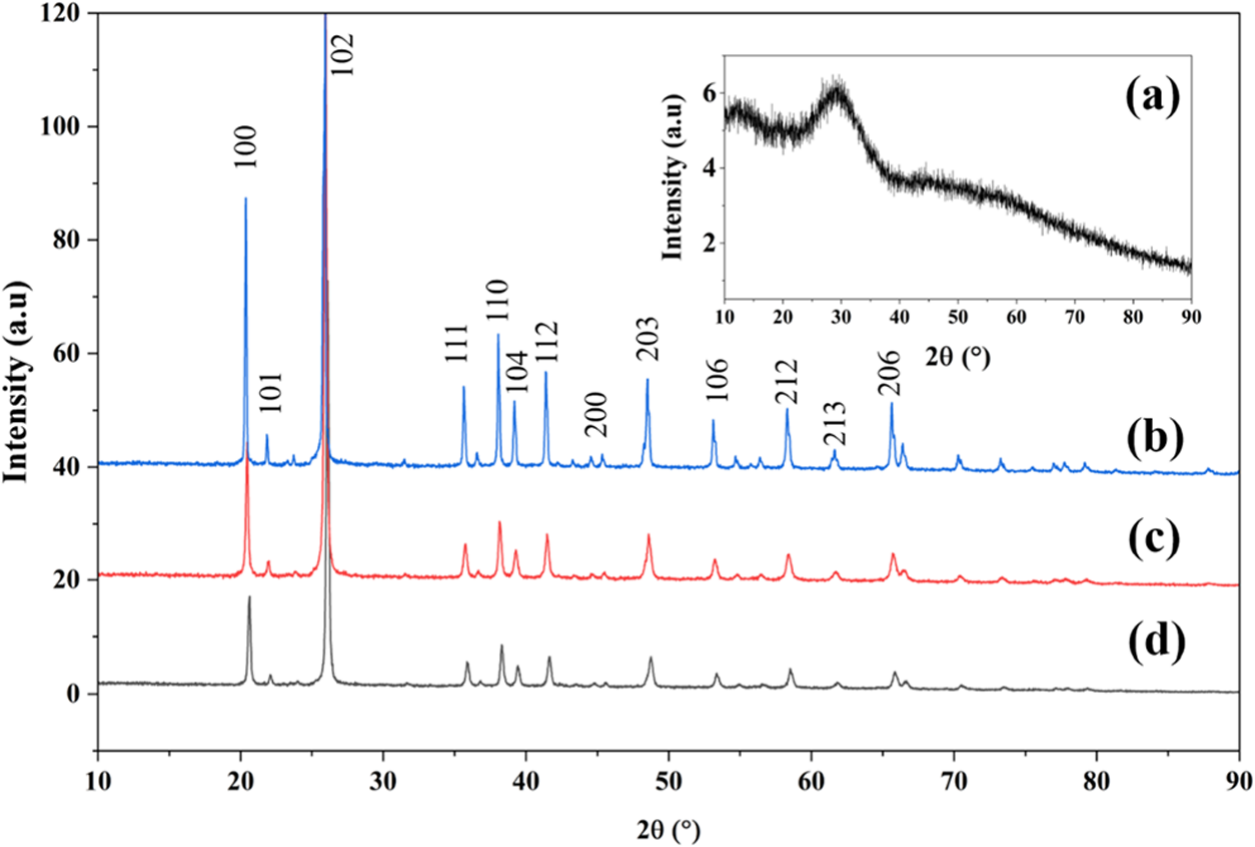
XRD patterns of (a) synthesized FePO_4_·2H_2_O, (b) synthesized anhydrous FePO_4_, (c) commercial FePO_4_ (batch 1), and (d) commercial FePO_4_ (batch 2).

After calcination at 720 °C, well-defined diffraction peaks are observed (Figure 3b–d). All reflections can be indexed to trigonal FePO_4_ (JCPDS card No. 29–0715), confirming the formation of crystalline anhydrous FePO_4_. The most intense peaks correspond to the (100) and (102) planes, indicating the development of long-range structural ordering after thermal treatment.

Scanning electron microscopy images show that each precursor maintained its characteristic micrometer-scale morphology after dehydration (Figure 2a,b); FP-B1 maintained its elongated, rod-shaped particles of 30 μm in length after calcination (Figure 2c,d); and FP-B2 retained its shape of large spherical agglomerates with varying diameters (10–50 μm) without changes after heat treatment (Figure 2e,f).

**Figure 2:**
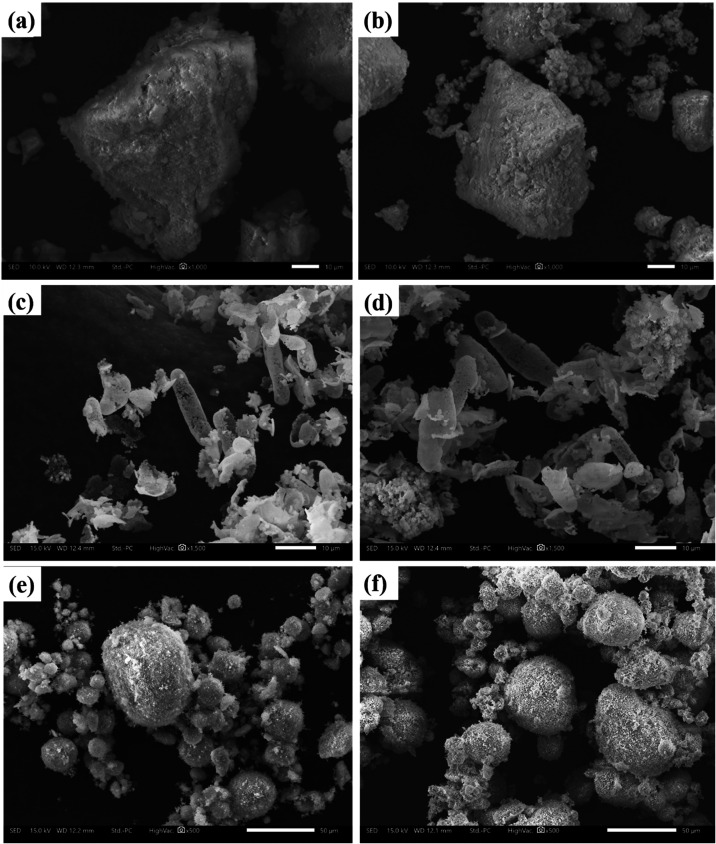
SEM images of (a) FePO_4_·2H_2_O; (b) FePO_4_; (c) Commercial FePO_4_·2H_2_O from batch 1; (d) commercial FePO_4_ from batch 1 after calcination; (e) FePO_4_·2H_2_O from batch 2; (f) FePO_4_ from batch 2 after calcination.

TG/DSC measurements were performed to evaluate the hydration and thermal stability of FePO_4_. Figure 3a shows the TG/DSC curves of synthesized FePO_4_·2H_2_O. As can be observed, a gradual mass loss occurs between 50 and 250 °C, corresponding to approximately 20%. The initial mass loss below 100 °C is attributed to the removal of physically adsorbed water, while the main mass loss between 100 and 250 °C is associated with the release of structural water from FePO_4_·2H_2_O. This value is close to the theoretical water content expected for the dihydrate phase, confirming the formation of FePO_4_ dihydrated.

**Figure 3:**
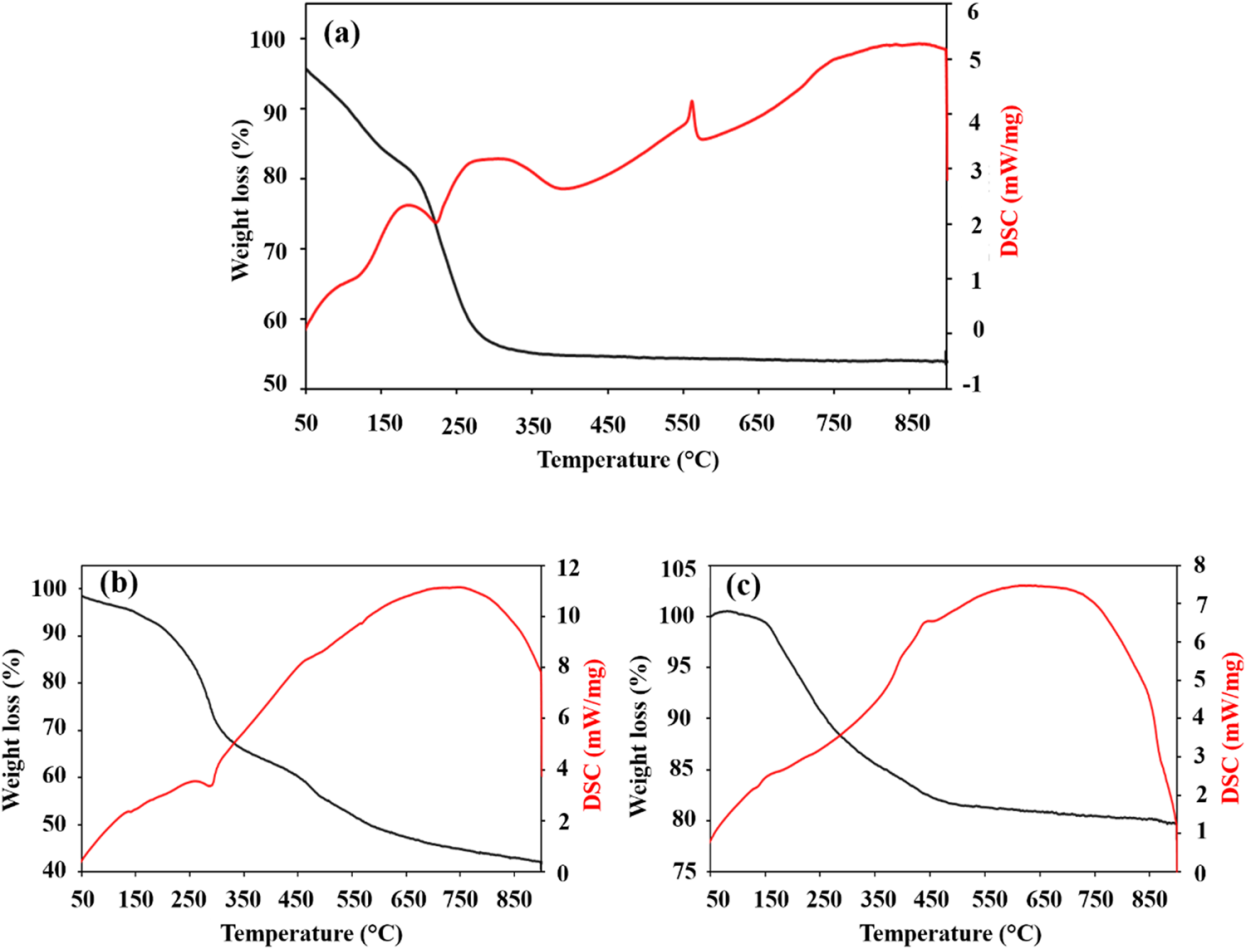
Thermal analysis of (a) FePO_4_·2H_2_O. (b) Precursor containing FePO_4_; and (c) precursor containing FeSO4, showing the TGA (black) and DSC (red) curves.

Above 250 °C, the TG curve becomes practically constant until 450 °C, indicating complete dehydration and the formation of anhydrous FePO_4_. An endothermic peak observed in the DSC curve at approximately 550 °C, without significant mass change, is attributed to a solid–solid phase transition of FePO_4_.

The thermal behavior of the LFP precursors was also evaluated and is shown in Figure 3b,c. In both cases, multiple mass loss events are observed. The main mass loss between 400 and 500 °C corresponds to the carbothermic reduction process leading to the formation of LiFePO_4_/C. Additional minor mass losses above 750 °C are related to the decomposition of residual carbon species and decarbonation reactions. These results indicate that the FePO_4_-based precursor exhibits a well-defined CO_2_ release step, followed by a thermally stable region before the formation of LiFePO_4_/C.

On the basis of the thermal behavior observed in the TG/DSC analysis, the synthesis of LiFePO_4_/C was carried out under the selected calcination conditions. Initially, the reactions for the formation of LFP were performed using FP-S and FS as iron sources at two different reaction times: 6 and 10 h. The XRD patterns obtained for these samples are listed in Figure 4.

**Figure 4:**
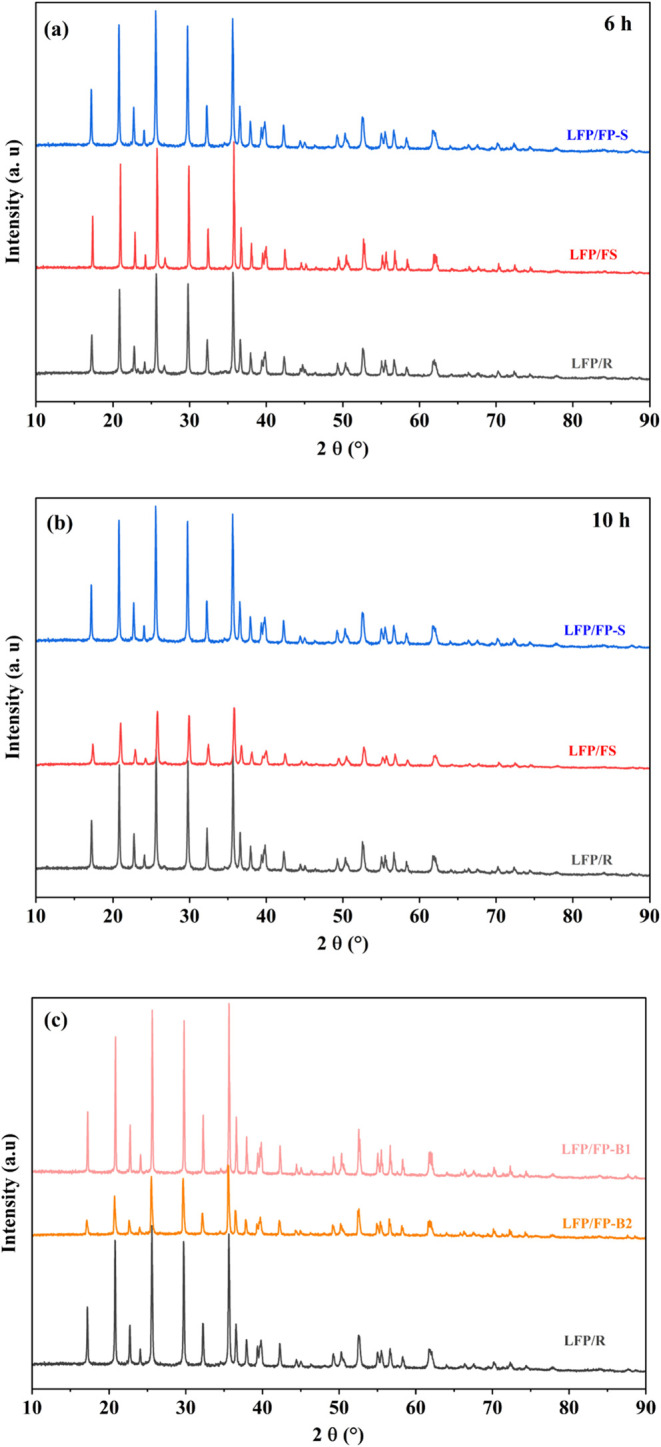
XRD patterns of (a) LiFePO_4_/C prepared using synthesized FePO_4_ (blue line) and FeSO_4_ (red line) precursors after 6 h and (b) 10 h, compared with commercial LiFePO_4_ (black line); (c) LiFePO_4_/C prepared using commercial FePO_4_ from batch 1 (light red line) and batch 2 (orange line) compared with commercial LFP (black line).

Figure 4 shows the X-ray diffraction patterns of LiFePO_4_/C samples synthesized using different iron precursors and reaction times. All diffraction peaks can be indexed to the orthorhombic structure of olivine LiFePO_4_ (space group *Pnma*, JCPDS fact sheet no. 81–1173). No secondary phase was detected within the instrument’s resolution limit.

A comparison between samples synthesized for 6 and 10 h reveals no significant differences in the position or relative intensity of the peaks, indicating that phase formation was already achieved after 6 h. Similarly, materials prepared from the FePO_4_ and FeSO_4_ precursors exhibit similar diffraction profiles.

Samples synthesized from commercial batches of FePO_4_ also show identical diffraction patterns, consistent with those of the commercial reference LiFePO_4_. The absence of additional peaks confirms the success of the carbothermic reduction and the formation of pure phase LiFePO_4_ in all cases. Carbon was not observed in the diffraction patterns, indicating an amorphous nature.

These results demonstrate that the types of precursors and the reaction times did not significantly affect the crystal structure of LiFePO_4_ under the studied conditions.

SEM micrographs (Figure 5a–d) show that, after 6 h, the precursor containing FS generated LFP with agglomerated particles with dimensions below 1 μm, while the precursor containing FP-S formed compact LFP aggregates with approximately 3–5 μm. After 10 h, the FS precursor presented micrometric agglomerates between 5 and 10 μm, while the FP-S precursor resulted in more uniform particles, distributed mainly between 2–6 μm, which indicated that increasing the synthesis time and the use of Fe^3+^ favored the production of a material with greater structural purity and more homogeneous morphology.

**Figure 5:**
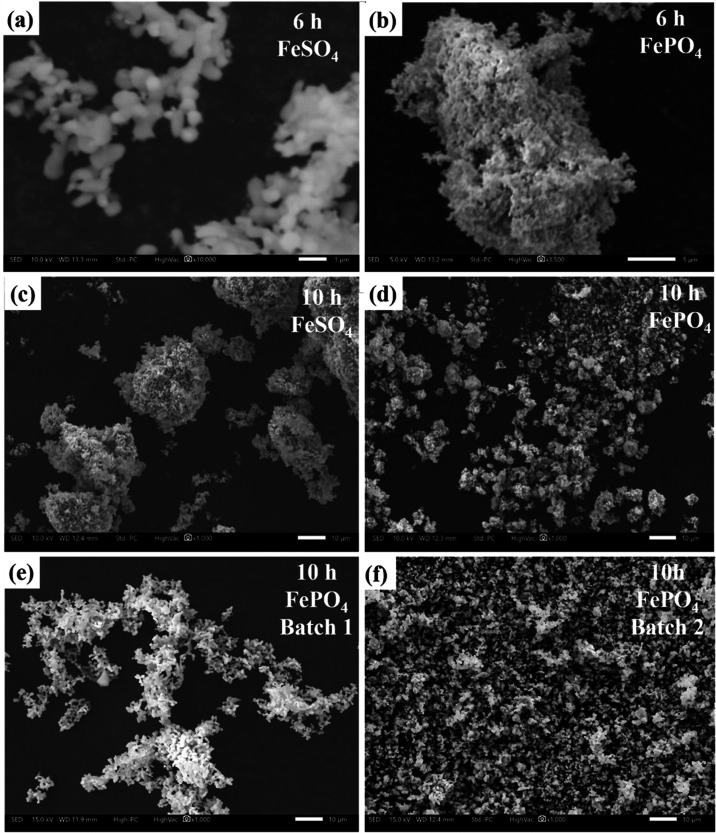
Scanning electron microscopy (SEM) images of LFP prepared with (a) FeSO_4_, 6 h, 10,000× magnification; (b) synthesized FePO_4_, 6 h, 3500× magnification; (c) FeSO_4_, 10 h, 1000× magnification; (d) FePO_4_, 10 h, 1000× magnification. (e) FePO_4_ from batch 1, 10 h, 1000× magnification; (f) FePO_4_ from batch 2, 10 h, 1000× magnification.

The particle size distribution profiles of LFP prepared from commercial FePO_4_-containing precursors (Supporting Figures S5–S6) further highlight the differences compared to the commercial reference LFP/R (Figure S7). LFP/FP-B2 exhibits a wider distribution, spanning approximately 3–15 μm, indicating greater heterogeneity compared to LFP/R, which shows a narrower and more symmetrical profile, predominantly in the 5–10 μm range. In contrast, LFP/FP-B1 shows a more confined distribution, centered mainly in the 5–10 μm range, closely matching the size range of LFP/R. These results are consistent with SEM observations, in which LFP/FP-B2 shows a more dispersed and heterogeneous particle arrangement, while LFP/FP-B1 exhibits more uniform clusters.

These morphological differences are further supported by the particle size distribution profiles (see Supporting Information, Figures S1–S7), which indicate distinct distribution patterns for each sample, including a bimodal profile for the FP-S-derived material. This feature may favor improved particle packing, as smaller particles can occupy interstitial spaces between larger ones, contributing to a more compact electrode structure.^16^

After confirming the formation of the olivine phase of LiFePO_4_ by XRD and evaluating the particle morphology, Raman spectroscopy and elemental analysis were performed to investigate the structural order of the carbon and variations in the composition of LFP through calcination (Table 2). Raman spectra can be viewed in Supporting Information S1.

**Table 2:** Influence of Calcination Time on Carbon Structural Order (*I*_D_/*I*_G_) and Elemental Composition of LiFePO_4_/C Samples

entry	sample/time of calcination	*I*_D_/*I*_G_ ^a^	carbon content (%)^b^	lithium content (%)^c^
1	LFP/FP-S (6 h)	0.65	1.2	4.34
2	LFP/FP-S (10 h)	0.64	1.1	4.40
3	LFP/FS (6 h)	0.68	6.4	3.40
4	LFP/FS (10 h)	0.76	4.4	4.07
5	LFP/FP-B1 (10 h)	1.08	1.39	4.25
6	LFP/FP-B2 (10 h)	0.99	0.60	4.31
7	LFP/R	1.16	3.13	4.22

a The *I*_D_/*I*_G_ ratio was calculated from Raman spectra by deconvolution of the D and G bands by using Origin peak fitting procedures.

b Carbon content was determined using a LECO CS844 carbon analyzer under an O_2_ flow.

c The lithium content was determined by inductively coupled plasma optical emission spectroscopy (ICP-OES) after acid digestion of the samples.

Raman analysis revealed significant differences in the structural orders of carbon among the samples. Although variations in carbon content were observed, no directly proportional relationship between the amount of carbon and the *I*_D_/*I*_G_ ratio was identified, particularly when the calcination time was taken into account.

For example, the LFP/FP-S samples showed low *I*_D_/*I*_G_ values at 6 and 10 h (0.64 and 0.65), even with relatively low carbon contents (1.2% and 1.1%), respectively, and no significant variation was observed when the calcination time was extended from 6 to 10 h, suggesting that the carbon coating reached a structurally stabilized state under these conditions. In contrast, the LFP/FS samples showed a decrease in the carbon content from 6.4% (6 h) to 4.4% (10 h), accompanied by an increase in the *I*_D_/*I*_G_ ratio from 0.68 to 0.76, indicating that prolonged calcination promoted carbon consumption and a greater degree of structural disorder.

Similarly, samples prepared with commercial iron phosphate, LFP/FP-B1, and LFP/FP-B1 calcined for 10 h showed *I*_D_/*I*_G_ values of 1.08 and 0.99, respectively, despite the relatively lower carbon content (1.39 and 0.6%), evidencing the formation of a more defective carbon structure in both cases.

The commercial reference sample (LFP/R) exhibited the highest *I*_D_/*I*_G_ ratio (1.16) with an intermediate carbon content (3.13%), reinforcing that the structural organization of carbon is not governed solely by carbon content but strongly depends on the precursor chemistry and the synthesis route. These results demonstrate that the structural organization of carbon is more strongly influenced by precursor chemistry and calcination conditions than by the total carbon content alone.

Figure 6 shows the rate capabilities of the prepared LFP electrodes at discharge current densities from 0.1C to 3.0C. The electrochemical measurements were performed in triplicate using independently assembled cells, demonstrating good experimental reproducibility. The commercial reference (LFP/R), used as received, showed 140 ± 3 mAh/g at 0.1C and maintained 100 ± 2 mAh/g at 3.0C, a typical profile for commercial materials that have undergone industrial processes.

**Figure 6:**
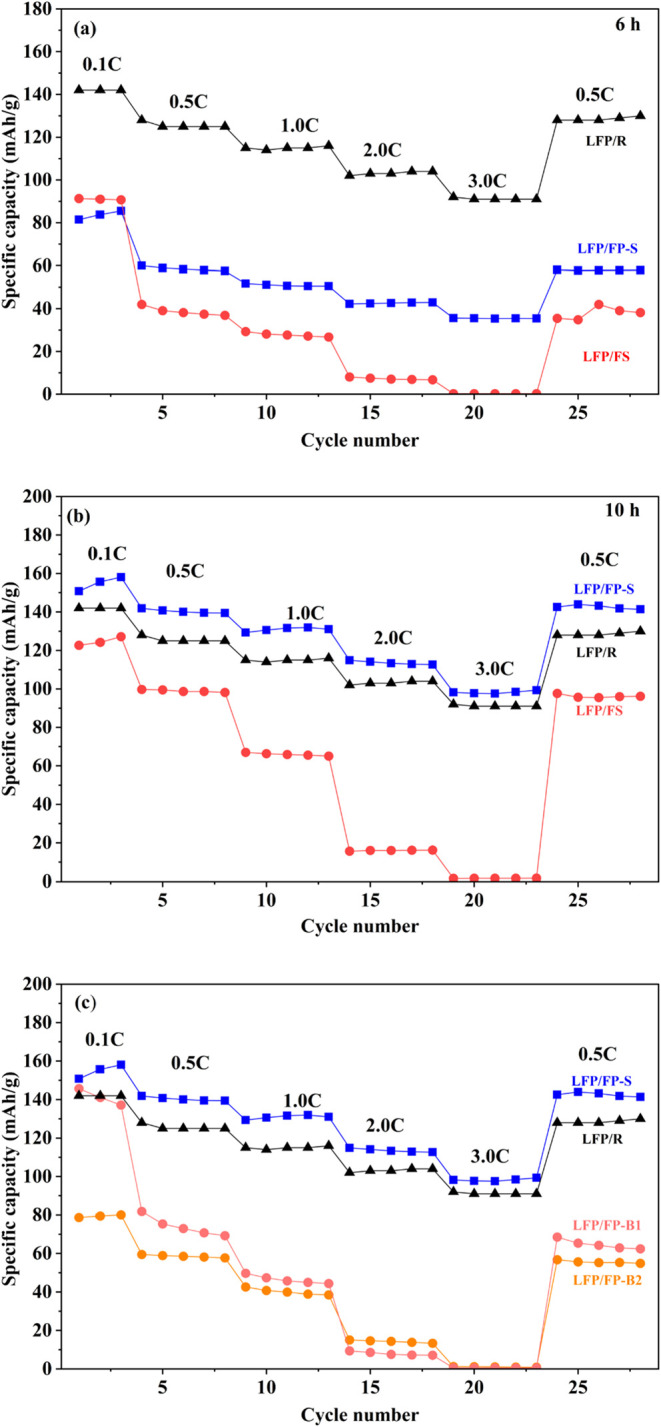
Rate capability at current densities ranging from 0.1C to 3.0C for (a) LFP/C samples synthesized for 6 h, (b) LFP/C samples synthesized for 10 h, and (c) LFP/C synthesized from commercial batches for 10 h.

For the synthesized LFP samples, the results of specific capacity show that calcination time was important in the synthetic route. At 6 h of calcination, both samples showed a rate capacity lower than the commercial reference. LFP/FP-S showed 80 ± 2 mAh/g at 0.1C and 40 ± 1 mAh/g at 3.0C, while LFP/FS started close to 90 ± 2 mAh/g at 0.1C, and showed a sharp decline, approaching zero capacity at 3.0C (Figure 6a).

This behavior is noteworthy considering the composition and Raman data, despite the high carbon content (6.4%) in the LFP/FS sample and its relatively low *I*_D_/*I*_G_ ratio (0.68) (Table 2, entry 3). In this case, the excess carbon may not effectively contribute to electron percolation and instead may introduce additional barriers to lithium-ion transport, especially if distributed as thicker or poorly connected domains. Such a configuration can increase ionic tortuosity and hinder Li^+^ diffusion through the electrode, particularly under high-discharge-rate conditions.

The LFP/FS sample also showed a poor performance at high discharge rates. In contrast, the LFP/FP-S sample, synthesized in 10 h, which contained only 1.1% carbon with an even lower *I*_D_/*I*_G_ ratio (0.64), exhibited better performance. These results indicate that the amount of carbon alone does not determine electrochemical performance and that the effectiveness of the conductive coating depends on the organization of the carbon and not just the content.

When the calcination time was increased to 10 h, a notable improvement was observed in LFP/FP-S, as its capacity increased from 90 ± 2 to 160 ± 3 mAh/g at 0.1C and remained close to 100 ± 2 mAh/g at 3.0C, very close to the performance of the commercial reference (Figure 6b). It is observed that this improvement occurred without significant changes in carbon content or *I*_D_/*I*_G_ ratio, as it remained at 0.64 and 1.1%, respectively, suggesting that longer calcination times primarily improved particle connectivity rather than the structural organization of the carbon coating.

For the LFP/FS calcined for 10 h, the low-rate capacity improved from 90 ± 2 to 120 ± 2 mAh/g; however, severe rate limitations persisted, with the capacity dropping again at 3C (Figure 6b). This phenomenon was observed precisely when there was a decrease in carbon content, from 6.4% to 4.4%, and an increase in the *I*_D_/*I*_G_ ratio, from 0.68 to 0.76, which shows a consumption of the carbon coating, due to the iron precursor already being at the correct oxidation number for the formation of LFP, and the increase in structural disorder, consequently resulting in an ineffective conductive coating for high currents.

In general, the electrochemical results show that, singly, neither a higher carbon content nor a lower *I*_D_/*I*_G_ ratio guarantees better performance at high discharge rates. LFP/FS, despite containing more carbon, showed low capacity at high rates and did not demonstrate significant improvement after an increase in calcination time. In contrast, LFP/FP-S, even with a much lower carbon content, showed significantly better performance after 10 h of calcination. Notably, this improvement occurred without substantial changes in carbon content or *I*_D_/*I*_G_ ratio, indicating that the improvement was not driven by an increase in graphitic order. The main difference lies in how the carbon coating was developed during heat treatment and in the particle size, which was smaller for LFP/FP-S. After 10 h, LFP/FP-S showed the best result; this calcination time was fixed and used in the experiment for comparison with commercial FePO_4_.

Figure 6c shows that both commercial batch of FePO_4_ used for the synthesis of LFP/FP-B1 and LFP/FP-B2 exhibited pronounced rate limitations compared to the synthesized LFP/FP-S. Although LFP/FP-B1 showed reasonable capacity at 0.1C (140 ± 3 mAh/g), its capacity decreased drastically with increasing C rate, approaching zero at 3.0C. LFP/FP-B2 exhibited an even lower initial capacity at 0.1C (80 ± 2 mAh/g) and severe rate limitation throughout the test. According to Raman and elemental analyses, LFP/FP-B1 contains 1.39% carbon with a high *I*_D_/*I*_G_ ratio of 1.08, while LFP/FP-B2 contains 0.6% carbon with an *I*_D_/*I*_G_ of 0.99, suggesting a more disordered carbon structure in both commercial materials compared to LFP/FP-S (*I*_D_/*I*_G_ = 0.65). These results indicate that the inferior high-rate performance of commercial FP-based samples is associated with a less effective conductive carbon network, regardless of carbon content.

SEM analysis further indicated that LFP/FP-S exhibited a smaller particle size than that of commercial FePO_4_ batches. Smaller particles are beneficial for electrochemical performance as they shorten Li^+^ diffusion paths and improve electrode kinetics, which may explain the superior rate capability observed for LFP/FP-S.^21,22^

Supporting Information Figure S15 shows the cycling stability of a half-cell assembled with the LFP/FP-S electrode, obtained from the synthesized FePO_4_ precursor, evaluated at 0.1C for 100 cycles. The cell exhibited a discharge capacity of 155 mAh/g after the cycles, which corresponds to a 98% retention of the initial capacity of 158 mAh/g, indicating minimal degradation. The Coulombic efficiency remained close to 100% throughout the test.

The effects of the iron precursor on the electrochemical performance of the cathode were further investigated by electrochemical impedance spectroscopy. EIS measurements were performed after the formation step, maintaining all cells at the same state of charge (50%). Nyquist impedance diagrams of the half-cells with cathodes of different LFPs are shown in Figure 7.

**Figure 7:**
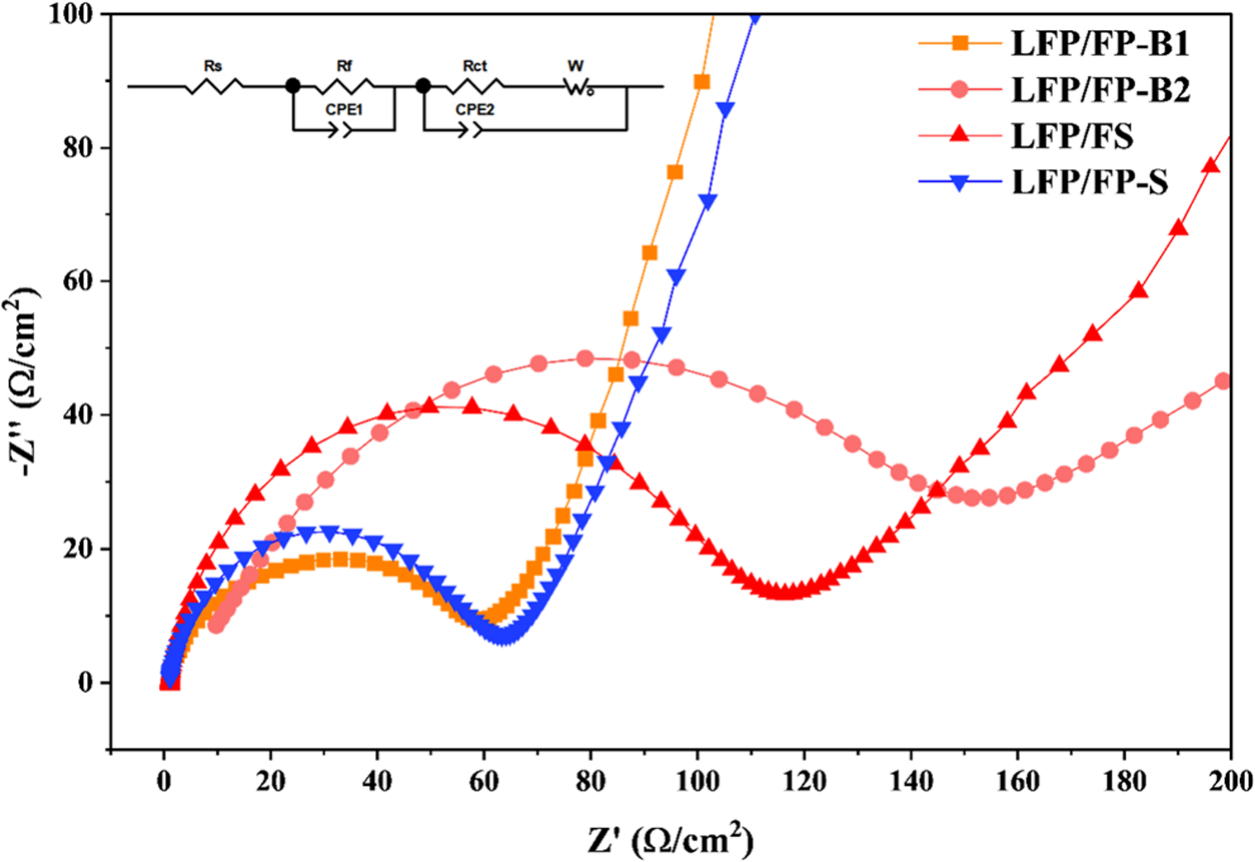
Nyquist plots of LiFePO_4_ electrodes prepared using different iron precursors. The inset shows the equivalent circuit used to fit the impedance data.

The impedance data obtained allowed for comparisons with the electrochemical behavior of the samples (Table 3), particularly when *R*_s_ and *R*_ct_ are considered together, rather than individually.^23–25^ The LFP/FP-S exhibits a low *R*_s_ value (1.22 Ω/cm^2^) combined with a similarly low *R*_ct_ value (63 Ω/cm^2^), resulting in reduced overall polarization and explaining its superior performance at high current rates (3C).^24,26^ Although the LFP/FP-B1 exhibits the lowest *R*_ct_ value among all samples (60 Ω/cm^2^), its higher *R*_s_ value (2.09 Ω/cm^2^) indicates less efficient electronic transport within the electrode, which can be detrimental under high current conditions where ohmic drops are more intense.^23,27^ On the other hand, LFP/FS exhibits the lowest *R*_s_ value (1.16 Ω/cm^2^), indicating good electronic conductivity; However, its significantly higher *R*_ct_ value (108 Ω/cm^2^) reveals that the interfacial charge transfer process is slower, which limits its rate capability, despite the favorable ohmic contribution.^28^ The worst performance was observed for LFP/FP-B2, which showed a drastic increase in both *R*_s_ (9.81 Ω/cm^2^) and *R*_ct_ (150 Ω/cm^2^), which is in good agreement with its lower specific capacity (80 mAh/g), indicating that the capacity of the cell is limited by poor interfacial charge transfer kinetics. The almost order-of-magnitude increase in *R*_s_ compared to LFP/FP-S indicates severe limitations in electron percolation and particle–particle contact, while the high *R*_ct_ value reflects slow interfacial kinetics.^29^ This combination leads to a pronounced widening of the semicircle in the Nyquist diagram and a strong displacement along the real axis, showing that both the resistive components of the volume and those of the interface are limiting factors. Under high charge/discharge rate conditions, such as 3C, these resistances translate directly into substantial voltage bias, reducing the effective capacitance.^26^

**Table 3:** Electrochemical Impedance Parameters of LiFePO_4_/C Electrodes Obtained from Different Iron Precursors

entry	sample	*R*_s_ (Ω/cm^2^)	*R*_ct_ (Ω/cm^2^)	σ (Ω cm^2^/s^1/2^)	*D*_Li_^+^ (cm^2^/s)
1	LFP/FP-S	1.22	63	9.0	8.35 × 10^–13^
2	LFP/FS	1.16	108	7.0	1.38 × 10^–12^
3	LFP/FP-B1	2.09	60	50	2.70 × 10^–14^
4	LFP/FP-B2	9.81	150	58	2.01 × 10^–14^

Considering that the iron precursor influences particle morphology, size distribution, and interparticle contact, variations in the electronic and ionic transport properties are expected. In particular, differences in particle organization and packing may impact lithium-ion diffusion and, consequently, the electrode kinetics. Therefore, to further evaluate these effects, the lithium-ion diffusion coefficient (*D*_Li_^+^) was calculated from the low-frequency region according to eq 1.^20^

1 $$D_{Li}^{+}=\frac{R^{2}T^{2}}{2A^{2}n^{4}F^{4}C^{2}\sigma^{2}}$$

where *R* is the gas constant (8.314 J/mol/K), *T* is the absolute temperature (298 K), *A* is the geometric area of cathode (2.01 cm^2^), *n* is the number of electrons per molecule during the electrochemical reaction (*n* = 1 according to Li^+^ ion intercalation/deintercalation reaction), *F* is the Faraday constant (96,485 C/mol), and *C* is the concentration of Li^+^ ions on film electrode (0.0114 mol/cm^3^). The Warburg factor (σ) is associated with *Z*′, according to eq 2.^20^

2 $$Z^{\prime }={(R}_{s}+R_{f}+R_{\mathrm{ct}})+\sigma \omega^{-1/2}$$

The diffusion behavior of lithium ions was evaluated from the linear relationship between *Z*′ and ω^–1/2^ in the low-frequency region, see Supporting Information (Figure S16). The slope of the graph corresponds to the Warburg coefficient (σ), which is inversely proportional to the diffusion coefficient of lithium, higher σ values indicate slower ion transport, whereas lower σ values correspond to faster diffusion kinetics. Samples made with commercial FePO_4_, LFP/FP-B1, and LFP/FP-B2 showed significantly higher slopes, indicating slow lithium-ion diffusion. In contrast, LFP/FS and LFP/FP-S showed much lower slopes, suggesting better diffusion kinetics. These results are consistent with the observed particle morphology, where smaller and more compact particles, due to the bimodal size distribution, decrease diffusion paths and facilitate ion transport.

As evidenced by the calculated *D*_Li_^+^ values (Table 3), clear differences in lithium-ion transport are observed between the samples. The LFP/FP-S and LFP/FS electrodes exhibited higher diffusion coefficients, while LFP/FP-B1 and LFP/FP-B2 showed significantly lower values, indicating slower lithium transport, with LFP/FP-B2 showing the lowest diffusion value, approximately 2.0 × 10^–14^ cm^2^/s (Table 3, Entry 4). This trend is consistent with the impedance results, particularly the Warburg coefficients, which reflect the diffusional contribution at low frequencies, confirming that lithium-ion transport limitations are a key factor governing the poor rate performance of LFP/FP-B2.

Lithium diffusion in LFP electrodes is complex as it involves contributions from solid-state diffusion within the active material, transport through the porous electrode, and migration in the electrolyte. In this context, since the electrode composition and test parameters were kept constant, the observed differences in the lithium-ion density (*D*_Li_^+^) are attributed solely to variations in transport behavior in the LFP samples.

Differences in lithium-ion transport are directly reflected in the electrochemical response, particularly under different current rates, where limitations due to the particle size become more evident.

The lower specific capacity observed for commercially available FePO_4_-derived LFP samples is likely associates to structural defects, specifically antisite disorder, within the synthesized material. In the olivine structure of LiFePO_4_ (space group *Pnma*), Li^+^ and Fe^2+^ ions occupy distinct crystallographic sites, namely M1 (Wyckoff 4a) and M2 (4c), respectively. However, considering the similar ionic radii of Li^+^ (0.76 Å) and high-spin Fe^2+^ (0.78 Å), partial exchange between these sites, known as antisite disorder, is thermodynamically feasible and has been reported in synthesized LFPs. The Fe^2+^ ions occupying the M1 site block the one-dimensional diffusion channels of Li^+^ along the *b*-axis, hindering ionic transport at high current rates.^30^

Numerous studies in the literature report the preparation of LiFePO_4_ by carbothermic reduction and related routes. Many different precursor systems are described: they originate from distinct iron sources, employ various carbon additives, and are subjected to different heat treatments. Particle sizes vary considerably, from nanometric powders to micrometric agglomerates. In these studies, electrochemical performance is frequently reported as discharge capacity at low C rates (0.1–0.2C). However, this parameter can be misleading if particle size, carbon content, and high-rate performance are not considered simultaneously (Table 4).

**Table 4:** Synthesis Methods, Morphology, and Electrochemical Performance of LiFePO_4_ Precursor Materials Obtained by Carbothermic Reduction^a^

type of precursor	synthesis method	particle size	observed morphology	discharge capacity 0.1C to 0.5C (mAh/g)	discharge capacity ≥ 1.0C (mAh/g)	refs
FePO_4_, Li_2_CO_3_, d-glucose, ball mill, 12 h, isopropanol	tubular furnace, 300 °C for 2 h, 700 °C for 10 h, Ar atmosphere	2 to 6 μm	uniform sphere	158 at 0.1C	100 at 3.0C	this work
NH_4_H_2_PO_4_, Li_2_CO_3_, Fe_2_O_3_, d-glucose, ball mill, 4 h, ethanol	tubular furnace, 300 °C for 2 h, 700 °C for 8 h, Ar atmosphere	1 to 2,5 μm	irregular sphere	159.3 at 0.1C	n. d	^35^
FePO_4_, Li_2_CO_3_, d-glucose, manual grinding in a mortar	tubular furnace, 650 °C for 9 h, N_2_ atmosphere	2,5 μm	uniform sphere	156.8 at 0.2C	144.1 at 1.0C	^31^
LiH_2_PO_4_, iron oxalate, d-gluconolactone	solvothermal method with ethylene glycol in a closed reactor: 270 °C for 10 h, 450 °C for 4 h in Ar/H_2_ (95:5)	nanoplates 1 μm wide and 20 to 30 nm thick	nanoplates	167.0 at 0.1C	n. d	^36^
NH_4_H_2_PO_4_, Li_2_CO_3_, Fe_2_O_3_, d-glucose, ball mill, 3 h, solvent-free	tubular furnace, 300 °C for 12 h, 700 °C for 12 h in N_2_	from 50 to 200 nm	clustered spheres	145.8 at 0.2C	n. d	^37^
				110 at 0.5C		
FePO_4_, Li_2_CO_3_, d-glucose, ball mill, 2 h, solvent-free	tubular furnace, 350 °C por 4 h e 650 °C por 12 h em N_2_	from 0.2 to 0.5 μm	elongated oval	150 at 0.2C	144 at 1.0C	^32^
FePO_4_, Li_2_CO_3_, sacarose, ball mil 2 h, solvent-free	tubular furnace, 700 °C por 16 h em N_2_	from 1 to 2 μm	clustered spheres	n.d	133 at 1.0C	^38^
					117 at 2.0C	
LiOH, FeSO_4_·7H_2_O, (NH_4_)_2_HPO_4_, sacarose, ball mill, 10 h, 250 rpm, solvent-free	tubular furnace, 600 °C por 6 h, N_2_/H_2_ (95:5)	from 100 to 250 nm	clustered spheres	n. d	148 at 1.0C	^39^
					142 at 2.0C	
					136 at 5.0C	
					114 at 10C	
FePO_4_, Li_2_CO_3_, d-glucose, ball mill, 6 h, ethanol	tubular furnace, 200 °C por 2 h, 450 °C por 5 h 700 °C por 10 h em Ar	1.0 μm	uniform sphere	160 in 0.2C	106 at 10C	^33^
LiOH, H_3_PO_4_, FeSO_4_, ascorbic acid, ball mill 350 rpm, 30 min, solvent-free	tubular furnace, 350 °C por 4 h, 700 °C por 10 h em Ar/H_2_ (95:5)	from 50 to 100 nm	uniform sphere	154 in 0.1C	95 at 10C	^34^
					43 at 10C	
Fe_2_O_3_, H_3_PO_4_, Li_2_CO_3_, d-glucose, water, 30 min ultrasound	tubular furnace, 650 °C por 10 h em Ar	from 100 to 200 nm	spherical	161 at 0.1C	119 at 10C	^40^
					93 at 20C	
LiOH, FeSO_4_, phytic acid, sacarose	solvothermal method with ethylene glycol in a closed reactor at 200 °C for 4 h, 650 °C for 6 h in an air atmosphere.	from 0.5 to 1.0 μm	spherical	166 at 0.1C	157 at 1.0C	^41^
					120 at 10C	

a n.d. = not determined.

For example, Wang et al.^31^ reported 144 mAh/g at 1.0C for uniformly spherical particles (∼2.5 μm), while Chen et al.^32^ obtained 144 mAh/g at 1.0C for particles between 0.2 and 0.5 μm (Table 3). Zhou et al.^33^ achieved 106 mAh/g at 10C for particles of approximately 1 μm, and Hsieh and Chien^34^ reported 95 mAh/g at 1.0C and 43 mAh/g at 10C for particles between 50 and 100 nm. Therefore, it is evident that, although high capacities at low rates are commonly achieved, maintaining performance at high C-rates remains a challenge and strongly depends on the morphology and efficiency of the conducting lattice.

To provide a more meaningful basis for comparison, performance at higher C rates should be evaluated together with the particle size and carbon content. According to these criteria, the LFP/FP-S material synthesized in this work exhibits a performance comparable to that of the systems summarized in Table 4. It provides 158 mAh/g at 0.1C and maintains 100 mAh/g at 3.0C despite having micrometric particles and a relatively low carbon content (1.1%).

When directly compared to LFP prepared from commercial FePO_4_ precursors under similar conditions, the synthesized precursor clearly demonstrates superior capacity at high discharge rates.

This difference can be attributed, at least in part, to the controlled morphology of the synthesized FePO_4_, which promotes smaller and more uniform particles than those observed for the commercial ones. Although the performance of LFP/FP-S does not exceed that of highly sophisticated nanostructured systems reported in the literature, it is achieved without an excessive carbon loading or complex synthetic strategies. These results indicate that precursor design is a decisive factor in LiFePO_4_ synthesis and that a controlled morphology can improve electrochemical performance at high rates.

Therefore, this study establishes that precursor-controlled morphology is a key parameter for optimizing lithium-ion transport and performance at high charge/discharge rates in LiFePO_4_, providing a simple and effective strategy to achieve high electrochemical performance without increasing the carbon content or relying on complex synthetic approaches.

## 4 Conclusions

In this work, LiFePO_4_/C was synthesized via a carbothermic route by using commercial and synthetic FePO_4_ precursors. We demonstrated that the precursor chemistry influences the microstructure development and, consequently, the final electrochemical performance. The use of FePO_4_ synthesized in our laboratories instead of commercial precursors resulted in superior capacity at high discharge rates, which was attributed to the controlled particle morphology, structural homogeneity, and the formation of a more efficient conductive carbon coating. It is worth highlighting that these improvements were achieved without increasing the carbon content of the synthesis or applying complex and expensive strategies. However, this study is limited to the synthesis conditions and specific precursors investigated, which did not include a detailed evaluation of the electrochemical aspects or scalability.

Future work should focus on scaling up the proposed method, other synthesis routes, and strategies to improve the performance of LFPs synthesized from commercial iron phosphate as well as investigating the assembly of other battery geometries such as cylindrical and prismatic ones.

## Supplementary Material



## Notes

Analyses of particle size distribution, Raman spectroscopy, cycling stability results, and Coulombic efficiency at 0.1C for LFP/FP-S, and electrochemical impedance spectroscopy analyses (Figures S1–S16) (PDF)

## References

[1] Gatta F. M., Geri A., Lamedica R., Lauria S., Maccioni M., Palone F., Rebolini M., Ruvio A. (2016). Application of a LiFePO4
Battery Energy Storage System to Primary Frequency Control: Simulations
and Experimental Results. Energies.

[2] del Valle, J. A. ; Anseán, D. ; Viera, J. C. ; Antuña, J. L. ; González, M. ; García, V. In Analysis of Advanced Lithium-Ion Batteries for Battery Energy Storage Systems, 2018 IEEE International Conference on Environment and Electrical Engineering and 2018 IEEE Industrial and Commercial Power Systems Europe (EEEIC/I&CPS Europe); IEEE, 2018; pp 1–6 10.1109/EEEIC.2018.8493934.

[3] Gutsch M., Leker J. (2022). Global Warming Potential of Lithium-Ion
Battery Energy Storage Systems:
A Review. J. Energy Storage.

[4] Padhi A. K., Nanjundaswamy K. S., Goodenough J. B. (1997). Phospho-olivines as Positive-Electrode
Materials for Rechargeable Lithium Batteries. J. Electrochem. Soc..

[5] Chandra G., Kashyap S. J., Sreedhara S. S., V Bulusu S., Ananthula V. V., R V., Rao T. N., Srinivasan A. (2023). Enhanced Stability
and High-Yield
LiFePO4/C Derived from Low-Cost Iron Precursors for High-Energy Li-Ion
Batteries. J. Energy Storage.

[6] Ramasubramanian B., Sundarrajan S., Chellappan V., Reddy M. V., Ramakrishna S., Zaghib K. (2022). Recent Development
in Carbon-LiFePO4 Cathodes for Lithium-Ion
Batteries: A Mini Review. Batteries.

[7] Rostami H., Valio J., Tynjälä P., Lassi U., Suominen P. (2024). Life Cycle of LiFePO4 Batteries:
Production, Recycling,
and Market Trends. ChemPhysChem.

[8] Qin Z., Li X., Shen X., Cheng Y., Wu F., Li Y., He Z. (2024). Electrochemical
Selective Lithium Extraction and Regeneration of
Spent Lithium Iron Phosphate. Waste Manage..

[9] Liu X., Sun L., Vu N. H., Linh D. T. H., Dien P. T., Hoa L. T., Lien D. T., Nang H. X., Dao V.-D. (2023). Synthesis of LiFePO4/Carbon/Graphene
for High-Performance Li-Ion Battery. J. Electroanal.
Chem..

[10] Zeng Y., Wang Y., Cai S., Li R., Zhou C., Wang C., Ma K., Song L., Yue H. (2024). All-Component
Recycling and Reuse Process for Spent LiFePO4 Cathodes. Ind. Eng. Chem. Res..

[11] Ni H., Liu J., Fan L.-Z. (2013). Carbon-Coated
LiFePO4–Porous Carbon Composites
as Cathode Materials for Lithium Ion Batteries. Nanoscale.

[12] Bai N., Xiang K., Zhou W., Lu H., Chen H. (2017). Hierarchical
Porous LiFePO4/Carbon Composite Electrodes for Lithium-Ion Batteries. Mater. Technol..

[13] Li W., Garg A., Le M. L. P., Ruhatiya C., Gao L., Tran V. M. (2020). Electrochemical
Performance Investigation of LiFePO4/C0.15-x
(X = 0.05, 0.1, 0.15 CNTs) Electrodes at Various Calcination Temperatures:
Experimental and Intelligent Modelling Approach. Electrochim. Acta.

[14] Coşkun E., Kurşun E., Yıldız B., Aşkar Y., Bahtiyar D., Aydınol M. K., Mavis B., Çınar-Aygün S. (2024). Size and Morphology
Controlled Polyol Synthesis of LiFePO4 Nanoparticles with Addition
of Organic Acid Combinations. Ceram. Int..

[15] Plummer L. K., Hutchison J. E. (2020). Understanding
the Effects of Iron Precursor Ligation
and Oxidation State Leads to Improved Synthetic Control for Spinel
Iron Oxide Nanocrystals. Inorg. Chem..

[16] Li P., Wang Y., Zhu L., Zhang K., Liu W., Chen T., Liu K. (2025). Particle Size
Grading Strategy for
Enhanced Performance of Lithium Iron Phosphate Cathode Materials. Crystals..

[17] Pierri E., Tsamouras D., Dalas E. (2000). Ferric Phosphate Precipitation in
Aqueous Media. J. Cryst. Growth.

[18] Lou W.-b., Zhang Y., Zhang Y., Zheng S.-l., Sun P., Wang X.-j., Qiao S., Li J.-z., Zhang Y., Liu D.-y., Wenzel M., Weigand J. J. (2021). A Facile Way to
Regenerate FePO4•2H2O Precursor from Spent Lithium Iron Phosphate
Cathode Powder: Spontaneous Precipitation and Phase Transformation
in an Acidic Medium. J. Alloys Compd..

[19] Martinez A. C., Dachraoui W., Murugesan R., Baudrin E., Demortière A., Becuwe M. (2022). Surface Modification of LiFePO4 Nanoparticles through
an Organic/Inorganic Hybrid Approach and Its Impact on Electrochemical
Properties. Colloids Surf., A.

[20] Stival U. A., Gallo I. B. C., Gonin C. F. N., Reis S. L., Grosso R. L., Kosctiuk J. B., Franchetti M. G. S., Leão B., Oliveira F. E. R., Souza A., Freitas H. R., Monteiro R. S., Parreira L. S., Berton M. A. C. (2023). Experimental
Challenges for Electrochemical
Evaluation of Cathodes in Lithium-Ion Battery Half-Cells. J. Energy Storage.

[21] Chang Y.-C., Peng C.-T., Hung I.-M. (2014). Effects of Particle
Size and Carbon
Coating on Electrochemical Properties of LiFePO4/C Prepared by Hydrothermal
Method. J. Mater. Sci..

[22] Wang J., Sun X. (2012). Understanding and Recent
Development of Carbon Coating on LiFePO4
Cathode Materials for Lithium-Ion Batteries. Energy Environ. Sci..

[23] Macdonald, J. R. ; Johnson, W. B. Fundamentals of Impedance Spectroscopy. In Impedance Spectroscopy; Wiley, 2018; pp 1–20 10.1002/9781119381860.ch1.

[24] Zhang S. S. (2006). The Effect
of the Charging Protocol on the Cycle Life of a Li-Ion Battery. J. Power Sources.

[25] Wei X., Zhang R., Li Y., Wang X., Wang N., Wang L., Zhang H., Ding F. (2026). Investigation of Impedance
Evolution in Li-Ion Batteries Following Lithium Plating and Online
Detection Methods. Electrochim. Acta.

[26] Xu B., Qian D., Wang Z., Meng Y. S. (2012). Recent Progress
in Cathode Materials Research for Advanced Lithium Ion Batteries. Mater. Sci. Eng., R.

[27] Wang J., Dong S., Lyu Y., Guo Z.-S. (2025). Understanding the
Effect of Particle Size and Size Standard Deviation on Lithium-Ion
Concentration and Discharge Behavior of Heterogeneous Electrode. Electrochim. Acta.

[28] Scanlan K., Manthiram A. (2025). Equations
and Electrochemical Methods for Measuring
the Interfacial Charge-Transfer Kinetics of Li-Ion Battery Active
Materials at High Current Densities. Electrochim.
Acta.

[29] Ebner M., Wood V. (2015). Tool for Tortuosity
Estimation in Lithium Ion Battery Porous Electrodes. J. Electrochem. Soc..

[30] Park K.-Y., Park I., Kim H., Yoon G., Gwon H., Cho Y., Yun Y. S., Kim J.-J., Lee S., Ahn D., Kim Y., Kim H., Hwang I., Yoon W.-S., Kang K. (2016). Lithium-Excess
Olivine Electrode for Lithium Rechargeable Batteries. Energy Environ. Sci..

[31] Wang L., Liang G. C., Ou X. Q., Zhi X. K., Zhang J. P., Cui J. Y. (2009). Effect of Synthesis
Temperature on the Properties of
LiFePO4/C Composites Prepared by Carbothermal Reduction. J. Power Sources.

[32] Chen C., Zhang Y., Wang F., Zou J. Z. (2011). Synthesis of LiFePO4/C
by Carbon Thermal Reduction Method and Its Electrochemical Properties. Adv. Mater. Res..

[33] Zhou D., Qiu X., Liang F., Cao S., Yao Y., Huang X., Ma W., Yang B., Dai Y. (2017). Comparison of the Effects of FePO4
and FePO4·2H2O as Precursors on the Electrochemical Performances
of LiFePO4/C. Ceram. Int..

[34] Hsieh, T. ; Chien, W. In Preparation of LiFePO4 Cathode Materials by Hydrothermal Method, Proceedings of 71st IASTEM International Conference; IASTEM, 2017; pp 24–28.

[35] Liu H.-p., Wang Z.-x., Li X.-h., Guo H.-j., Peng W.-j., Zhang Y.-h., Hu Q.-y. (2008). Synthesis and Electrochemical
Properties
of Olivine LiFePO4 Prepared by a Carbothermal Reduction Method. J. Power Sources.

[36] Saravanan K., Balaya P., Reddy M. V., Chowdari B. V. R., Vittal J. J. (2010). Morphology
Controlled Synthesis of LiFePO4/C Nanoplates for Li-Ion Batteries. Energy Environ. Sci..

[37] Liu A., Liu Y., Hu Z., Gao G., Xu Y., Lei L. (2011). Electrochemical
Performance of LiFePO4/C Synthesized by Solid State Reaction Using
Different Lithium and Iron Sources. J. Phys.
Chem. Solids.

[38] Gao J., Li J., He X., Jiang C., Wan C. (2011). Synthesis
and Electrochemical
Characteristics of LiFePO4/C Cathode Materials from Different Precursors. Int. J. Electrochem. Sci..

[39] Sarkar S., Mitra S. (2014). Carbon Coated Submicron
Sized-LiFePO4: Improved High Rate Performance
Lithium Battery Cathode. Energy Procedia.

[40] Liu R., Chen J., Li Z., Ding Q., An X., Pan Y., Zheng Z., Yang M., Fu D. (2018). Preparation of LiFePO_4_/C Cathode Materials via a Green Synthesis Route for Lithium-Ion
Battery Applications. Materials.

[41] Li Y., Wang L., Zhang K., Yao Y., Kong L. (2021). Optimized
Synthesis of LiFePO4 Cathode Material and Its Reaction Mechanism during
Solvothermal. Adv. Powder Technol..

