# Supplementary material for: Comparison of Synthesized Microstructured and Commercial FePO4 as Precursors for High-Performance LiFePO4/C Cathode Materials
Source: ACS Omega. 2026 Jul 11;11(29):43682–93. doi: 10.1021/acsomega.6c02734 (PMC13425511; doi:10.1021/acsomega.6c02734)
Supplement: Supplementary file 1 [file ao6c02734_si_001.pdf]

Supporting Information

Comparison of Synthesized Microstructured and Commercial $FePO_4$ as Precursors for High-performance $LiFePO_4$/C Cathode Materials

Leandro Alves dos Santos [a,b*], Elton Torres Zanoni [a], Gabriella M. V. Dias [a], Sofia Sestito Dias [a], Francisca E. R. Oliveira [a], George C. Santos [c], Frederico F. Taves [c], Tatiana A. Souza [c], Rodrigo V. Queiroz [c], Heverson R. Freitas [a], Guilherme Panini [a], Adler de Souza [a], Agne R. Carvalho-Jorge [a], Marcos A. C. Berton [a]

[a] SENAI Innovation Institute in Electrochemistry, Curitiba, 81350-010, Paraná, Brazil

[b] Department of Chemistry, Federal University of Paraná, Polytechnic Center, P.O. Box 19032, Curitiba, 81531-980, Paraná, Brazil

[c] Centrais Elétricas do Brasil, Rio de Janeiro, 20071-003, Rio de Janeiro, Brazil

*Corresponding Author

E-mail address: leandroalvsantos@sistemafiep.org.br

Keywords: Iron phosphate precursor; Microstructured FePO4; Carbothermal synthesis; Particle size control; Rate capability; LiFePO4/C cathodes

Supplementary text 1. Determination of particle size distribution

The particle size distribution was determined by dynamic light scattering (DLS) using a Microtrac S3500 instrument. The analysis was performed with the powder suspension using deionized water as the dispersing medium. The analyzed powder materials were placed in a beaker with deionized water and kept in an ultrasonic bath (40 kHz) for 5 min.

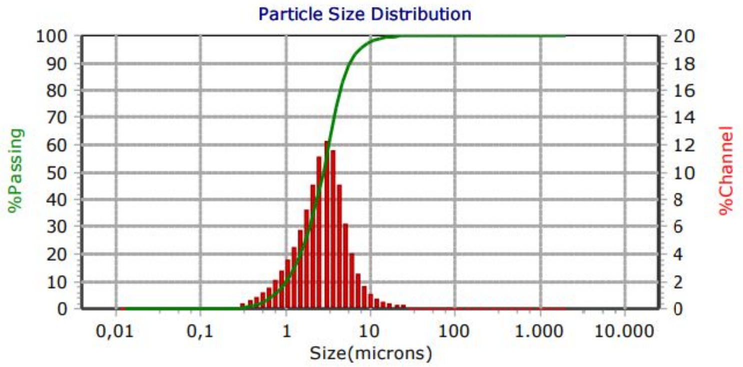

Supplementary Fig. S1. Particle size distribution of LFP/FP-S (reaction time of 6 h).

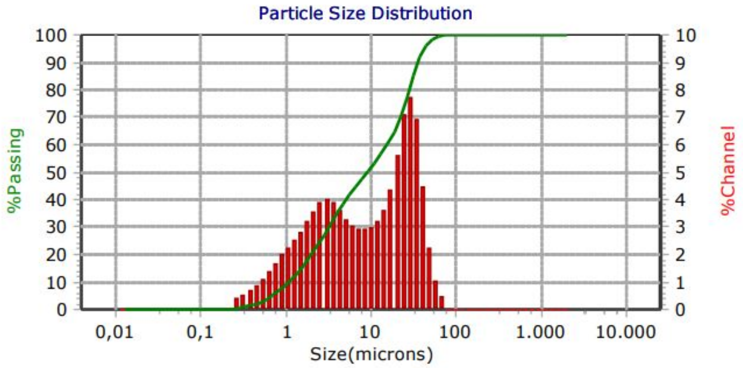

Supplementary Fig. S2. Particle size distribution of LFP/FP-S (reaction time of 10 h).

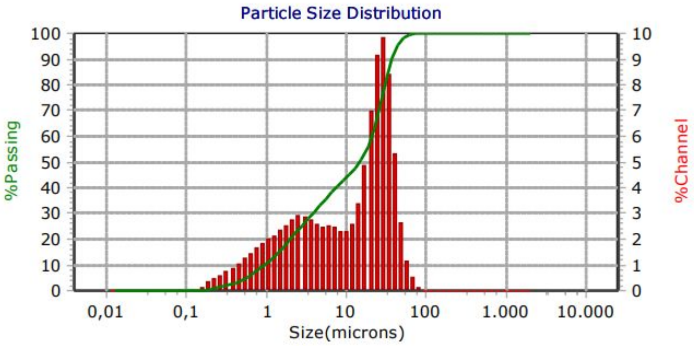

Supplementary Fig. S3. Particle size distribution of LFP/FS (reaction time of 6 h).

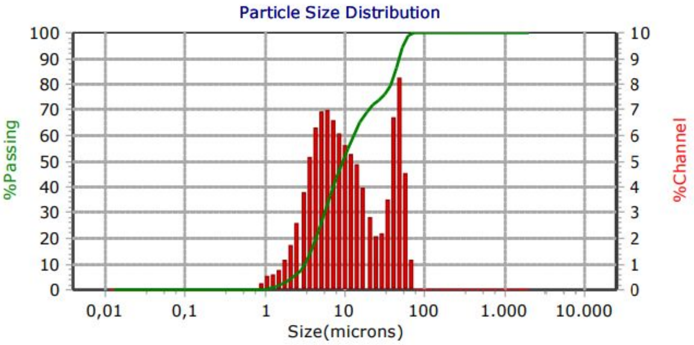

Supplementary Fig. S4. Particle size distribution of LFP/FS (reaction time of 10 h).

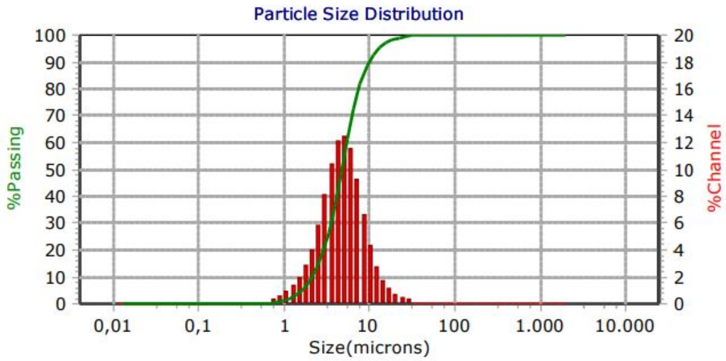

Supplementary Fig. S5. Particle size distribution of LFP/FP-B1 (reaction time of 10 h).

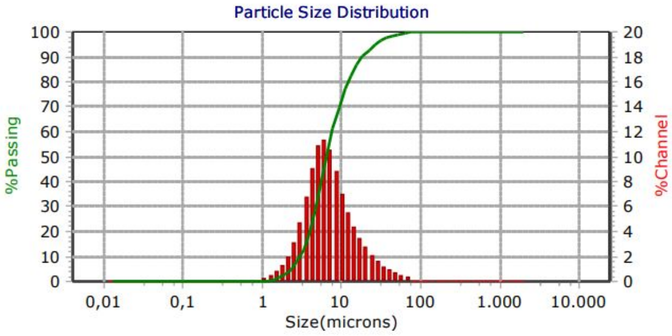

Supplementary Fig. S6. Particle size distribution of LFP/FP-B2 (reaction time of 10 h).

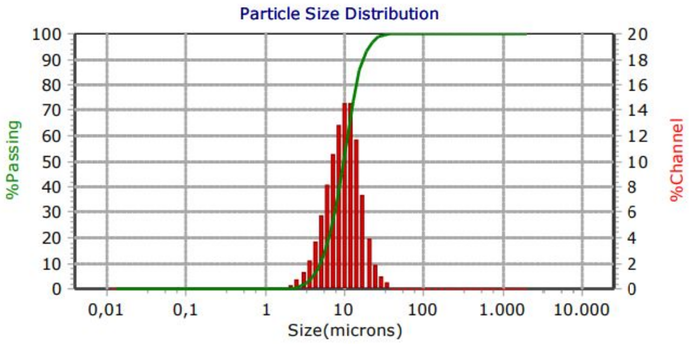

Supplementary Fig. S7. Particle size distribution of LFP/R.

Supplementary text 2

Raman

The Raman spectra revealed significant differences in the carbon layer formation in the two LFPs synthesized from Fe^{2+} and Fe^{3+} (Supplementary Figures 8-14) The bands at 1301 cm^{-1} (D) and 1585 cm^{-1} (G) confirm the presence of amorphous and graphitic carbon, respectively. Sample LFP/FS presented higher intensities in these bands, reflecting a higher residual carbon content (4.4%) compared to sample LFP/FP-S (1.1%). This difference is directly related to the reducing role of D-glucose during calcination. In the system containing Fe^{3+} ($FePO_4$), a significant portion of D-glucose is consumed in its reduction to Fe^{2+} in $FeSO_4$, which leads to the oxidation of the precursor carbon to CO and CO_2, decreasing the amount of solid carbon in the LFP particle. In the Fe^{2+} system, this reduction step is not necessary, allowing a larger fraction of D-glucose to be converted to carbon, which favors the formation of a thicker conductive layer. Furthermore, the I_D/I_G ratio indicates that the deposited carbon is mostly amorphous, which, despite its lower ordering, contributes positively to the material's electrical conductivity.

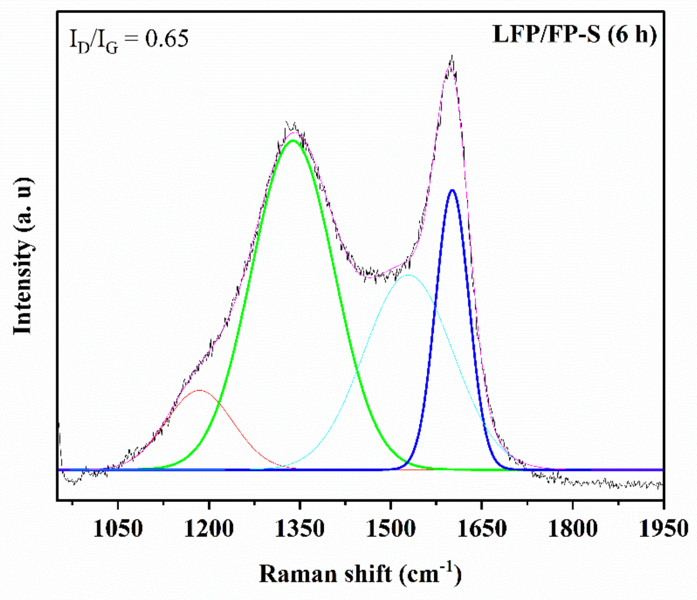

Supplementary Fig. S8. Raman spectrum of LFP/FP-S (reaction time of 6 h) with peak deconvolution.

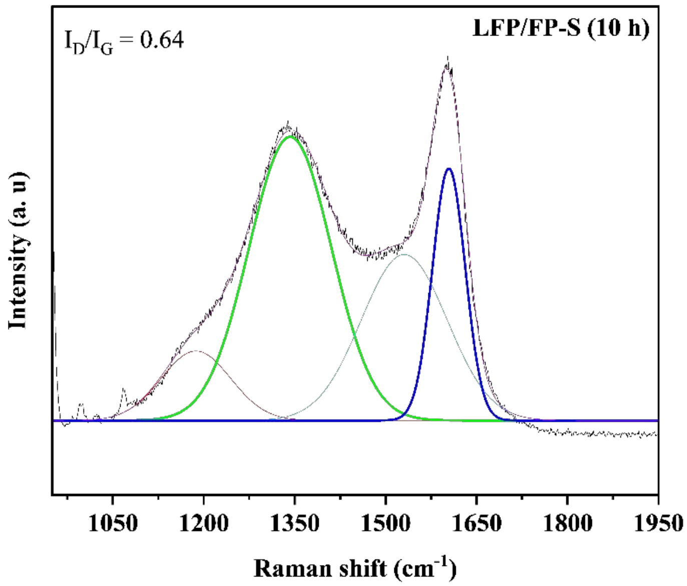

Supplementary Fig. S9. Raman spectrum of LFP/FP-S (reaction time of 10 h) with peak deconvolution.

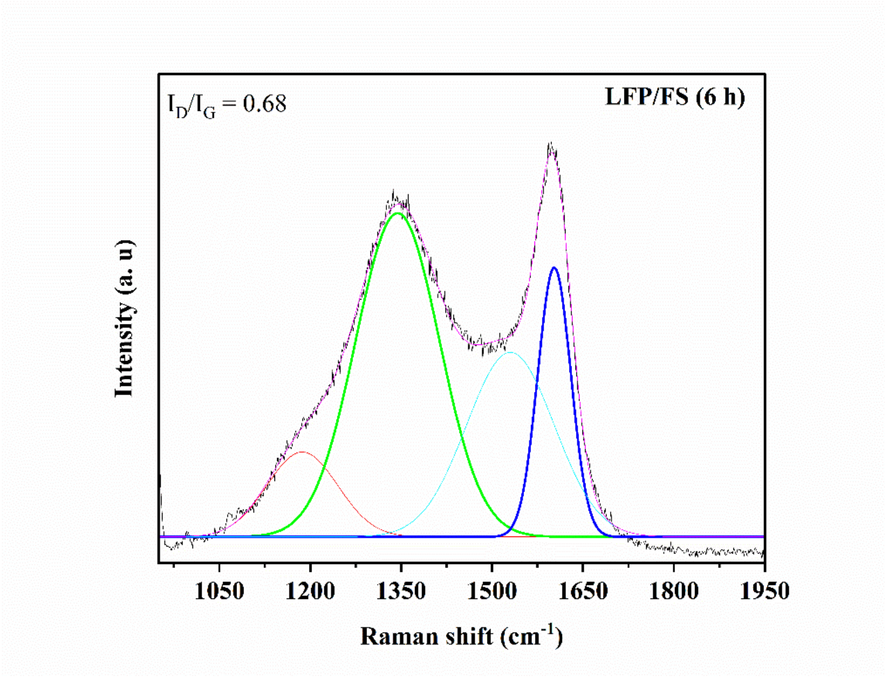

Supplementary Fig. S10. Raman spectrum of LFP/FS (reaction time of 6 h) with peak deconvolution.

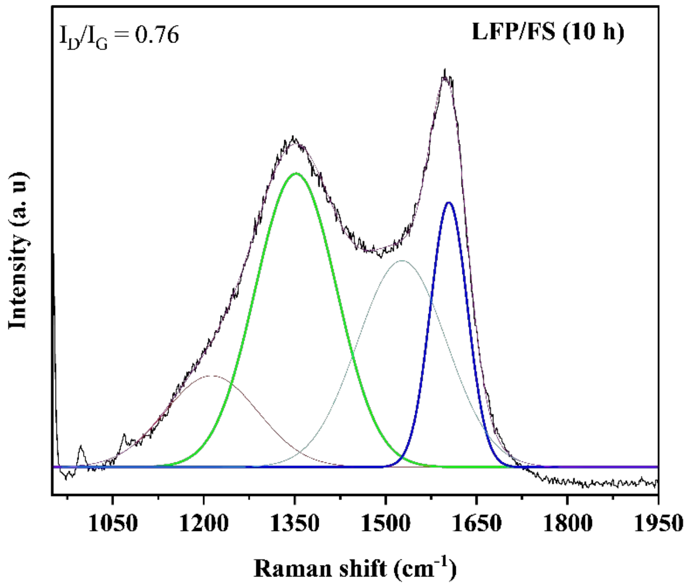

Supplementary Fig. S11. Raman spectrum of LFP/FS (reaction time of 10 h) with peak deconvolution.

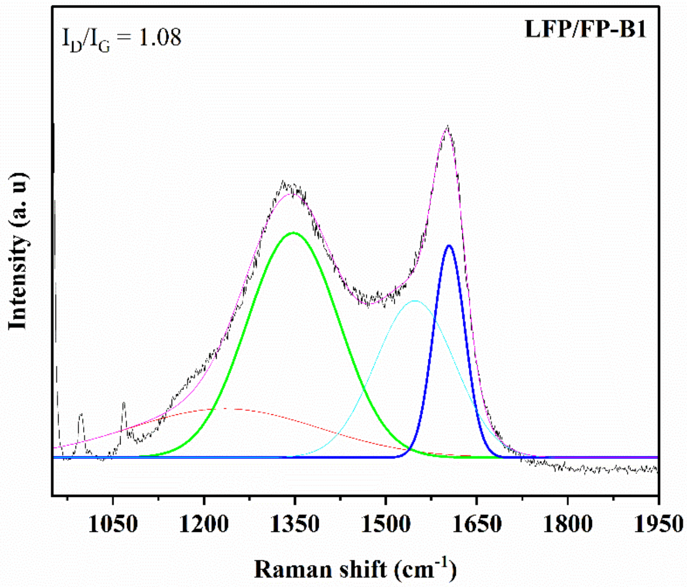

Supplementary Fig. S12. Raman spectrum of LFP/FP-B1 (reaction time of 10 h) with peak deconvolution.

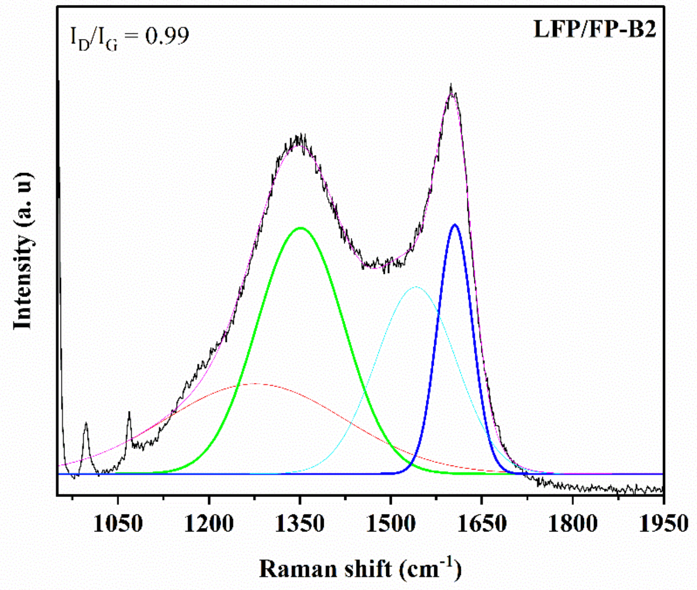

Supplementary Fig. S13. Raman spectrum of LFP/FP-B2 (reaction time of 10 h) with peak deconvolution.

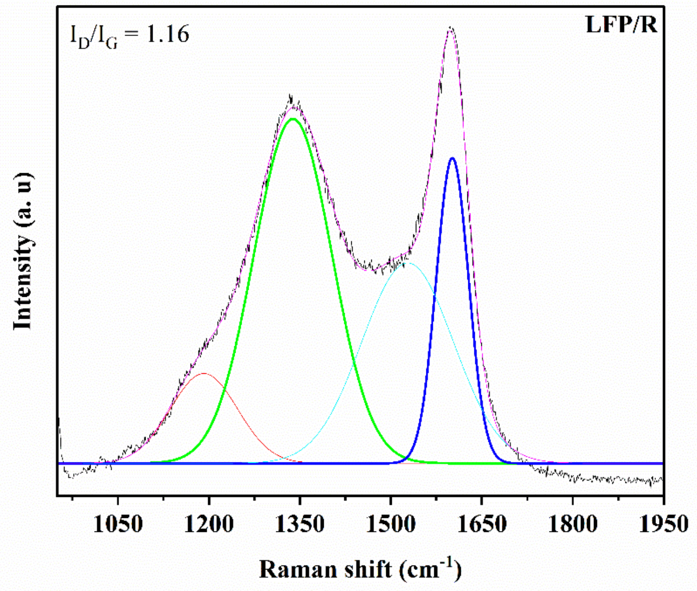

Supplementary Fig. S14. Raman spectrum of LFP/R (reaction time of 10 h) with peak deconvolution.

Supplementary text 3

Cycling stability and coulombic efficiency under 0.1 C of LFP/FP-S

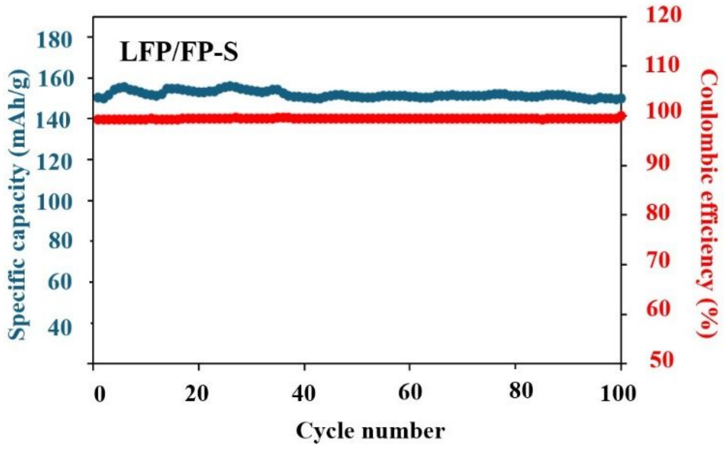

Supplementary Fig. S15. Cycling performance and coulombic efficiency of LFP/FP-S over 100 cycles.

Supplementary text 4

Electrochemical impedance spectroscopy

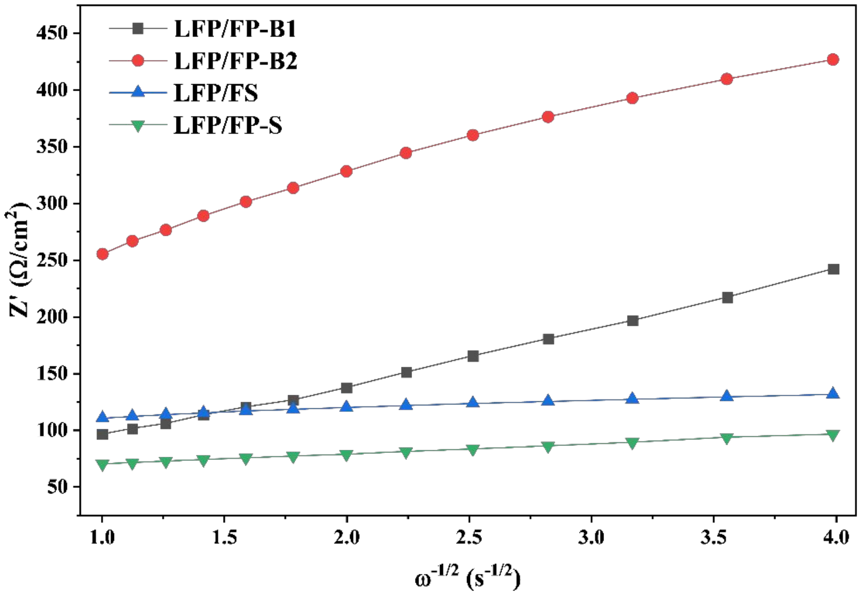

Supplementary Fig. S16. Linearized low-frequency Warburg plot: Z’ as a function of $\omega^{-1/2}$ for LFP samples.
